# A *POLE* P286R‐Mutated Hepatoid Adenocarcinoma of the Lung Showing Substantial Response to Immune Checkpoint Inhibition

**DOI:** 10.1002/cnr2.70661

**Published:** 2026-09-08

**Authors:** Aleksey Chernev, Alina Kaurtseva, Ruslan Abasov, Rafael Enikeev, Nina Gegelia, Gulnara Abdrakova, Natalia Usman, Alexander Druy, Grigory Raskin

**Affiliations:** ^1^ Dr. Sergey Berezin Medical Institute St. Petersburg Russian Federation; ^2^ Dmitry Rogachev National Medical Research Center of Pediatric Hematology, Oncology and Immunology Moscow Russian Federation

**Keywords:** hepatoid adenocarcinoma of the lung, immunotherapy, mismatch repair deficiency, *POLE* p.Pro286Arg mutation

## Abstract

**Background:**

Hepatoid adenocarcinoma of the lung (HAL) is a rare form of lung cancer with histological and morphological properties of hepatocellular carcinoma, characterized by an aggressive course of the disease and unfavorable prognosis. Given the rarity of the pathology and the lack of unambiguous therapeutic protocols, descriptions of clinical cases of HAL deserve attention.

**Case:**

We describe a case of metastatic HAL in a 61‐year‐old patient, harboring a mismatch repair deficiency associated with the loss of PMS2 protein expression. Molecular‐genetic study identified several oncogenic variants including *PMS2* p.Arg421Ter, *PMS2* p.Arg107Trp, and *POLE* p.Pro286Arg and confirmed their somatic status. The laboratory findings clinically justified the use of immunotherapy with pembrolizumab, which afforded stabilization of the disease according to computed tomography data and complete tumor response according to morphological examination data.

**Conclusion:**

The presented case highlights the room for immunotherapy in the highly aggressive HAL. The *POLE* p.Pro286Arg missense mutation has been attributed as a predictor for immune checkpoint inhibition efficacy.

## 1 Background

Hepatoid adenocarcinoma of the lung (HAL) is a rare variety of lung cancer accounting for < 0.1% of lung adenocarcinomas [1] and 2.3% of hepatoid adenocarcinomas of various localization [2]. For the first time described in 1990, HAL is found primarily in Asian populations [3, 4]. The median age of manifestation is 63, and the male‐to‐female ratio was 8:1. The tumor shows predilection for the upper lobes—particularly the right upper lobe—accounting for 61%–72% of cases. Most patients present at advanced stages, with Stage IV disease in 53%–68% of cases, and CNS metastases at diagnosis in as many as 40% [5, 6].

Morphologically, HAL corresponds to hepatocellular carcinoma with alpha‐fetoprotein production observed in most cases. The diagnostic hallmark is a tumor composed of hepatocyte‐like cells arranged in a trabecular or solid pattern with eosinophilic cytoplasm and centrally placed nuclei, exactly recapitulating hepatocellular carcinoma morphology [6]. The immunohistochemical (IHC) profile of HAL consistently includes positivity for HepPar‐1, CK7, CK18, MOC‐31, and HEA125. AFP is elevated in 53%–76% of patients at diagnosis, reflecting tumor activity but not functioning as an independent prognostic factor. TTF‐1 frequently shows cytoplasmic staining (distinct from nuclear positivity in conventional lung adenocarcinoma). CK7 and CEA positivity with TTF‐1 nuclear negativity effectively distinguishes HAL from metastatic hepatocellular carcinoma [6, 7].

The molecular landscape of HAL comprises *TP53* mutations in the majority of cases. *STK11*, *SMARCA4*, *CDKN2A*, and *CDK8* aberrations are recurrently being described [8, 9]. With 191 cases of primary HAL described and summarized so far, the rarity of the pathology, the unfavorable prognosis, and the lack of standardized therapeutic algorithms necessitate further search for effective clinical options, which potentially include immunotherapeutic approaches [7].

## 2 Case

**FIGURE 1:**
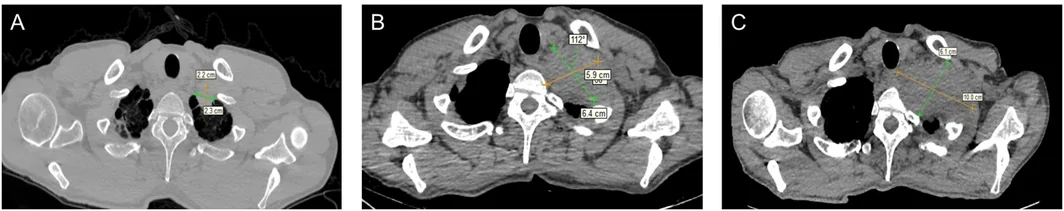
CT scans of the primary tumor lesion in the S1/2 of the left lung during the course of the disease prior to immune checkpoint inhibition: (A) before treatment (23 × 22 mm); (B) after multiple successive regimens of chemotherapy (112 × 64 mm); and (C) after the palliative course of external beam radiotherapy (100 × 61 mm).

A 61‐year‐old male underwent computed tomography (CT) of the chest for bullous pulmonary emphysema in June 2020, revealing a mass in the S1–S2 segments of the left lung (15 × 19 mm), which increased to 22 × 23 mm after 7 months (Figure 1A). A similar lesion (11 × 15 mm) was also noted in the S6 segment of the right lung. No biopsy/specific treatment was performed due to the patient's refusal. One year later (November 2021), the pathological mass at the apex of the left lung invaded the anterior chest wall and soft tissues of the neck, with destruction of the body of the 1st rib.

An ultrasound‐guided aspiration biopsy of the left lung lesion was performed, resulting in a diagnosis of metastasis of a poorly differentiated adenocarcinoma, probably hepatocellular carcinoma. The pathological lesion in the right lung was considered a metastasis and was not biopsied. Successive lines of chemotherapy with cisplatin and docetaxel (both 75 mg/m^2^, 2 cycles, progression), irinotecan (35 mg/m^2^, four infusions, progression), gemcitabine (1000 mg/m^2^, 1 cycle of three infusions, progression), cisplatin and etoposide (both 100 mg/m^2^, 2 cycles, progression) did not achieve disease control, and continued tumor growth in the upper lobe of the left lung was observed (Figure 1B). After unsuccessful attempts at systemic treatment, the patient was admitted to the Dr. Sergey Berezin Medical Institute where they underwent a palliative course of external beam radiotherapy to the area of the primary lesion and metastases of adenocarcinoma in the lymph nodes of the left supraclavicular region to a total focal dose of 40 Gy in single focal doses of 8 Gy (September, 2022). The radiation therapy had a limited effect as the primary tumor lesion shrank by 29%. CT performed after its completion revealed a lesion in the upper lobe of the left lung (S1–S2, Pancoast cancer), with signs of involvement of the left subclavian and vertebral arteries, and destruction of the first rib on the left and left portions of the C7 and Th1 vertebrae (Figure 1C).

**FIGURE 2:**
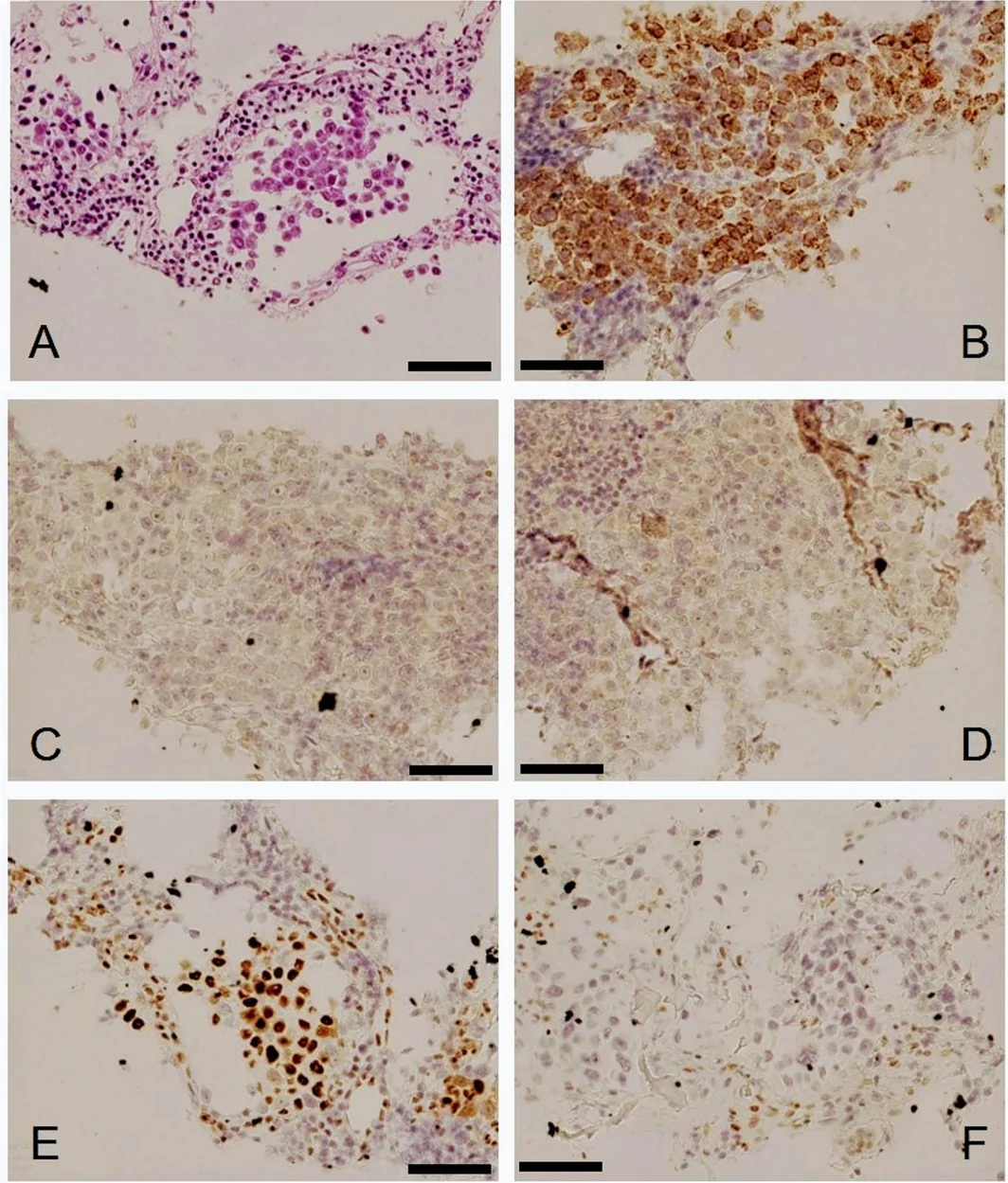
Histology (hematoxylin–eosin, A) and immunohistochemistry (positive for HepPar—B, negative for p63—C and TTF1—D, retained MSH6 expression—E and loss of PMS2 expression– F) of the tumor material acquired by ultrasound‐guided aspiration biopsy. Magnification ×400. Scale bar corresponds to 50 μm.

**FIGURE 3:**
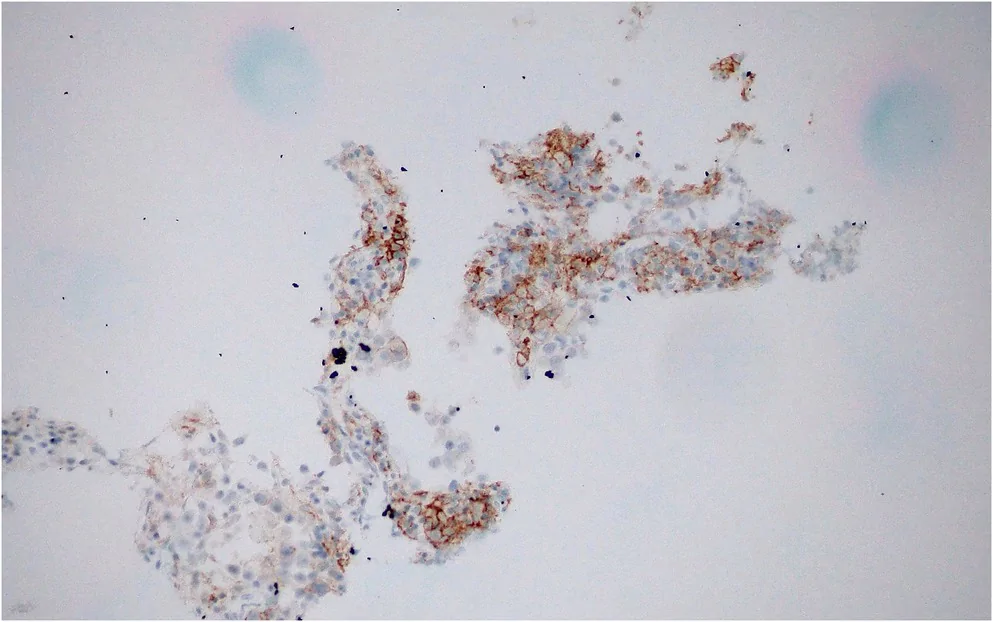
The expression of PD‐L1 (clone 22C3). Magnification ×400.

Reassessment of the primary biopsy material in the Center of Laboratory Medicine, Dr. Sergey Berezin Medical Institute, revealed epithelioid cells forming nested clusters (Figure 2A), expressing cytokeratins 7 and 19, HepPar (Figure 2B), BRG1, and lacking expression of p63 (Figure 2C), TTF1 (Figure 2D), and CDX2. The MSH6 expression was retained (Figure 2E), while PMS2 expression was lost (Figure 2F). Expression of EpCAM (MOC31/HEA125) and alpha‐fetoprotein (including its serum levels) was not evaluated. The expression of PD‐L1 (clone 22C3, Agilent, USA) was detected in 2% of tumor cells and 15% of immune cells (Figure 3). It is important to note that the size of tumor specimen was substantially restricted (less than 100 cells), resulting in the borderline scope of PD‐L1 expression. However, the histological structure and immunophenotype of the tumor were found to correspond to hepatoid adenocarcinoma with signs of mismatch repair deficiency (based on the PMS2 expression loss) and PD‐L1 expression (Combined positive score was 17). Molecular genetic studies performed using allele‐specific PCR and FISH methods revealed no *EGFR*, *BRAF*, and *KRAS* mutations or *ALK* and *ROS1* gene rearrangements.

**FIGURE 4:**
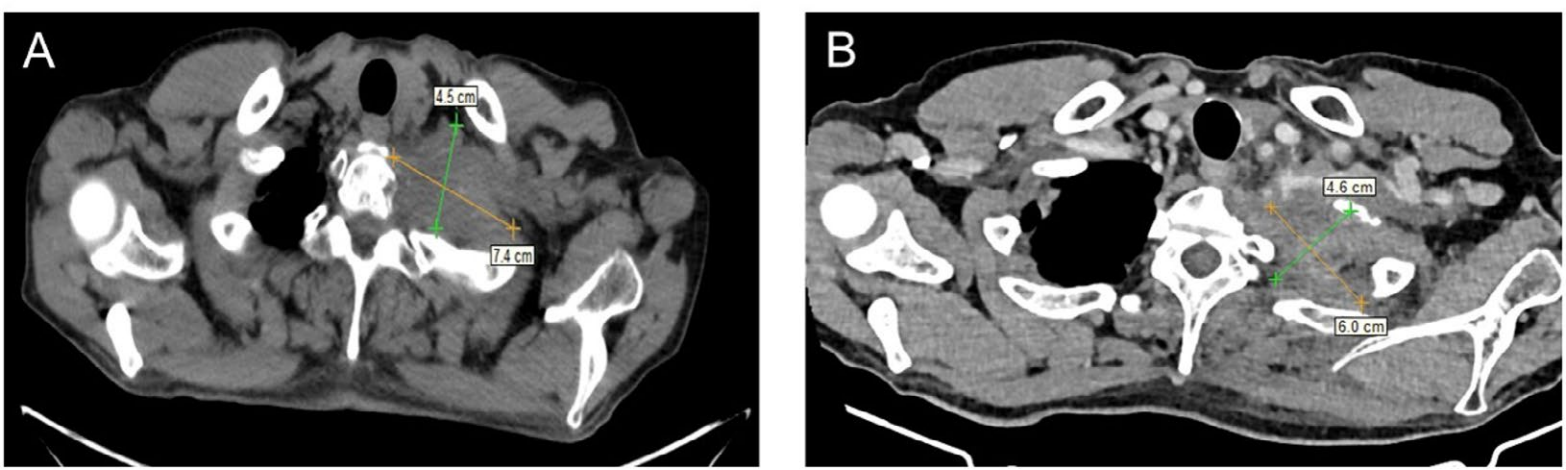
CT scans of the primary tumor lesion in the S1/2 of the left lung at immune checkpoint inhibition: (A) after three infusions of pembrolizumab (74 × 45 mm) and (B) the best response after 18 infusions of pembrolizumab (60 × 46 mm).

Immunotherapy with pembrolizumab (200 mg on Day 1 of a 21‐day cycle) was commenced in October 2022 (since 1 month after the completion of radiation therapy) as a salvage therapy, based on the PMS2 expression loss and PD‐L1 expression. Three cycles of pembrolizumab afforded a 45.4% regression of the apical tumor in the left lung (to 74 × 45 mm, Figure 4A), while enlargement of the S6 lesion size in the right lung, which had remained stable since detection. The pembrolizumab therapy was continued.

The IHC sign of mismatch repair deficiency prompted a molecular genetic investigation of its causes. High‐throughput sequencing of the constitutional DNA from peripheral blood leukocytes revealed no pathogenic or potentially pathogenic variants in genes of the mismatch repair system. Also, the study of DNA isolated from peripheral blood leukocytes using MLPA technology (SALSA MLPA Probemix ME011, MRC‐Holland, the Netherlands) revealed no deletions or aberrant methylation patterns of the *MLH1*, *MSH2*, *PMS2*, and *MSH6* genes, thereby excluding Lynch syndrome.

DNA isolated from the histological material was subject to panel high‐throughput sequencing with amplicon target enrichment. The median sequencing depth for the region of interest was 1515×. The analysis revealed multiple somatic oncogenic genetic variants (Table 1) including *PMS2* p.Arg421Ter, *PMS2* p.Arg107Trp, and *POLE* p.Pro286Arg. The two *PMS2* variants explained the loss of PMS2 protein expression, and the mutation in the exonuclease domain of Pol‐ε explained the potential accumulation of somatic mutations in the tumor cell genome.

**TABLE 1:** Genetic variants revealed by high‐throughput sequencing of DNA isolated from the tumor tissue.

Gene	Transcript ID	Identified genetic variant		Variant allele frequency (VAF), %
		Gene coding sequence	Amino acid sequence of the protein	
*PMS2*	NM_000535.7	c.1261C>T	p.Arg421Ter	14
*PMS2*	NM_000535.7	c.319C>T	p.Arg107Trp	19
*POLE*	NM_006231.4	c.857C>G	p.Pro286Arg	13
*ARID1B*	NM_001374828.1	c.3592C>T	p.Arg1198Ter	11
*PTEN*	NM_000314.8	c.959 T>C	p.Leu320Ser	13
*TP53*	NM_000546.6	c.844C>T	p.Arg282Trp	11
*TP53*	NM_000546.6	c.523C>T	p.Arg175Cys	12

Control examinations performed after pembrolizumab cycles 9, 15 and 18 indicated the lack of dynamics in the size of the tumor lesion at the apex of the left lung. The preserved spread into the soft tissues of the neck and the destruction of the 1st rib on the left, the body of C7 and the left transverse process of Th1 were evident (Figure 4B). Trepanobiopsy of the lesion in the left supraclavicular region, performed under ultrasound control after 18 cycles of pembrolizumab (November 2023), comprised fragments of loose connective tissue with foci of necrosis; the lack of tumor cells within the examined material corresponded to a complete tumor response. The pathological lesion in the S6 segment of the right lung substantially decreased in volume by 77.1% during the immunotherapy.

The treatment with pembrolizumab is well tolerated and is not accompanied with any adverse events. To the date of the last follow‐up examination (June 2026), the patient has received 61 cycles of pembrolizumab, with stabilization of the underlying disease and a good quality of life for 3.7 years. The entire timeline of events is presented in the Table 2.

**TABLE 2:** The timeline of the patient's course of the disease and treatment interventions.

Dates	June 2020	January 2021	October 2021	November 2021	February 2022	April 2022	May 2022	July 2022	September 2022	October 2022
Treatment or intervention				Biopsy	Cisplatin, docetaxel	Irinotecan	Gemcitabine	Cisplatin, etoposide	Radiation therapy	Pembrolizumab
S1/2 left lung lesion	19 × 15 mm	22 × 23 mm	48 × 47 mm	62 × 44 mm	64 × 54 mm	64 × 53 mm	81 × 62 mm	112 × 64 mm	100 × 61 mm	95 × 51 mm
S6 right lung lesion	15 × 11 mm	21 × 15 mm	17 × 14 mm	17 × 14 mm	14 × 11 mm	14 × 11 mm	15 × 8 mm	15 × 11 mm	13 × 8 mm	21 × 10 mm

Dates	December 2022	February 2023	May 2023	July 2023	September 2023	November 2023^a^	January 2024	Subsequent follow‐up every 3 months		June 2026
Treatment or intervention	Pembrolizumab									
S1/2 left lung lesion	74 × 45 mm	76 × 50 mm	55 × 47 mm	55 × 47 mm	66 × 65 mm	46 × 60 mm	46 × 60 mm	46 × 60 mm	46 × 60 mm	46 × 60 mm
S6 right lung lesion	23 × 20 mm	16 × 10 mm	17 × 11 mm	15 × 7 mm	15 × 7 mm	15 × 7 mm	15 × 7 mm	15 × 7 mm	15 × 7 mm	15 × 7 mm

^a^ Trepanobiopsy of the left supraclavicular lesion.

## 3 Discussion

To our knowledge, there are no descriptions in the literature of HAL with a pathogenic variant in the exonuclease domain of DNA polymerase ε, which could lead to the accelerated accumulation of somatic mutations and subsequent tumor immunogenicity. The identified genetic variant *POLE* p.Pro286Arg, widely described in endometrial and colorectal cancer samples, has been associated with high tumor mutational burden [10, 11]. Generally, the *POLE* exonuclease domain mutations are known to be a mechanism of DNA polymerase ε proofreading deficiency and subsequent accumulation of passenger genetic variants [12]. In the recently published paper, A. Vemuri et al. highlight extremely high tumor mutation burden in cases of endometrial carcinoma with specifically *POLE* p.Pro286Arg mutation ranging from 78 to 277 (median 169) mutations per megabase [13]. In colorectal cancer with *POLE* exonuclease domain mutations, tumor mutation burden achieved 115 (range 61–216) mutations per megabase [14]. In the studied clinical case of HAL, the tumor reveals a mutational profile similar to POLE‐mutated endometrial cancer, including *PMS2*, *ARID1B*, *PTEN*, and *TP53* variants. The deficiency of exonuclease activity of Pol‐ε determines the secondary mutation‐driven inactivation of the MMR genes and/or *TP53* mutations in the “Multiple‐Classifier” endometrial cancer; accordingly, such cases belong to the group of POLE‐mutated tumors [15, 16, 17, 18]. Similarly, in the described case of HAL, a nucleotide substitution in a *POLE* “hot spot” could explain the accelerated mutation process in tumor cells, eventually bringing on the mutations determining the loss of PMS2 and two amino acid substitutions in the DNA‐binding domain of p53. Indeed, the loss of PMS2 expression can be mechanistically explained by revealed biallelic somatic mutations, one of which leads to the protein truncation, and the second determines amino‐acid substitution in the highly conservative position. Despite we were unable to determine the tumor mutation burden in accuracy due to inappropriate panel size (2.9 kilobases), the hypermutated phenotype is highly anticipated because of the spectrum of revealed genetic variants and previously published evidence of *POLE* p.Pro286Arg substitution.

The most comprehensive genomic profiling of HAL contains 32 NGS‐characterized cases that identified 31 recurrently mutated genes including *TP53, STK11, SMARCA4, CDKN2A, KRAS, CDK8*, and *EPHA5* [7]. The single case of HAL harboring *FAT1* c.3940 T>A substitution and copy number loss was described [9]. The tumor mutation burden is generally low in reported HAL cases accounting from 1.69 mut/Mb in a case with *FAT1* mutation to 8.67 mut/Mb in a case with *STK11/TP53/NOTCH1* co‐mutations [9, 19]. Most reported HAL tumors are microsatellite‐stable, generally limiting the population expected to respond to checkpoint inhibition via the MSI‐high/dMMR pathway.

*STK11* mutations which are recurrently present in HAL have profound implications for immune microenvironment composition. In lung adenocarcinoma broadly, *STK11* inactivation creates an immunosuppressive, “immune desert” tumor microenvironment characterized by reduced density of infiltrating cytotoxic CD8+ T‐cells, near‐absent TCF1+ stem‐like CD8+ T‐cells, a neutrophil‐enriched rather than lymphocyte‐enriched microenvironment, and activated angiogenesis and epithelial‐mesenchymal transition. The co‐mutation of *KRAS* and *STK11* observed in the subset of HAL is the most immunosuppressive genomic combination across all types of lung cancer [7, 8, 20].

Despite the obvious advantages of the integrated multi‐omics approach for prediction of the immunotherapy effectiveness, HAL‐specific multi‐omics data are essentially non‐existent due to the tumor's extreme rarity. No published study has performed integrated genomic, transcriptomic, epigenomic, and proteomic profiling specifically in HAL. Because HAL cases can harbor both immunosuppressive (*STK11* and *SMARCA4*) and immunogenic (*TP53*) mutations, blanket immune checkpoint inhibition predictions are unreliable without individual profiling.

Due to the rare incidence of HAL, there are no standardized approaches to the treatment of this tumor, while its biological aggressiveness dictates the need to find effective treatment methods, for example, immunotherapy with immune checkpoint inhibitors. A HAL‐focused meta‐analysis by Deng et al. provides data on the use of immunotherapy in 16 patients [7]. In a single case among this sample, immunotherapy was prescribed in connection with mismatch repair deficiency in a patient with Lynch syndrome causing the loss of expression of PMS2 and MLH1 proteins in the tumor tissue. Despite the lack of PD‐L1 expression in the tumor or its reactive microenvironment, a partial response was obtained with durvalumab [21]. Another case of successful use of immunotherapy in HAL was associated with increased mutational burden of the tumor co‐existing with an inactivating variant in *SMARCA4* [22]. For the rest of the analyzed cases of using immunotherapy for the treatment of HAL, no information on the presence of predictive markers was available [7].

In the current case, the tumor mutational burden could not be determined with accuracy due to the extremely small amount of tumor substrate and the use of a small panel for sequencing. Still, the identified *POLE* p.Pro286Arg substitution accompanied by multiple oncogenic variants affecting other genes suggests a potentially elevated mutational burden. The inferred mismatch repair deficiency (as defined by the loss of PMS2 protein expression) and the presence of PD‐L1 positive cells provided a clinical rationale for immunotherapy. The use of pembrolizumab halted the fulminant progression of the chemoresistant tumor and afforded the ongoing disease control. Considering initiation of the immune checkpoint blockade after 1 month since radiation therapy, the synergistic effect of these treatment modalities could be assumed. Moreover, morphological examination of the residual tumor substrate reveals complete replacement of the pathological lesion with connective tissue, suggesting substantial effect of immunotherapy in the described case of HAL. It should be noted that the right lung lesion considered as a metastatic site demonstrated significant reduction upon pembrolizumab therapy but was not biopsied and evaluated histologically. Thus, systemic treatment response is probable; however, it cannot be stated definitely.

## 4 Conclusion

The article presents the first description of HAL harboring the *POLE* p.Pro286Arg mutation interpreted as the cause of the apparently elevated mutational burden of the tumor. The loss of exonuclease activity probably has led to secondary *PMS2* inactivation and mismatch repair deficiency aggravated by two missense mutations in the *TP53* gene. The identified genetic markers, along with the evidence on PD‐L1 expression by cells of the neoplasm, determined the rationale for and the actual efficacy of immunotherapy, with a pronounced effect resulting in the complete morphological response of the residual tumor. According to the presented evidence and similar cases published previously, the presence of DNA polymerase ε proofreading deficiency due to *POLE* somatic mutation could be considered as a tissue‐agnostic predictor of high tumor immunogenicity and immune checkpoint blockade effectiveness.

## Author Contributions

**Aleksey Chernev:** investigation, data curation, writing – original draft. **Grigory Raskin:** conceptualization, writing – review and editing, data curation. **Rafael Enikeev:** investigation. **Alina Kaurtseva:** investigation. **Nina Gegelia:** investigation. **Natalia Usman:** writing – original draft. **Alexander Druy:** writing – original draft, conceptualization, investigation, data curation, writing – review and editing. **Gulnara Abdrakova:** investigation. **Ruslan Abasov:** software, investigation.

## Funding

The authors have nothing to report.

## Ethics Statement

The investigation and publication of the data was approved by Dr. Sergey Berezin Medical Institute Ethics board.

## Consent

Informed voluntary consent for the publication of diagnostic, treatment, follow‐up details including medical images was obtained from the patient.

## Conflicts of Interest

The authors declare no conflicts of interest.

## Data Availability

The data that support the findings of this study are available on request from the corresponding author. The data are not publicly available due to privacy or ethical restrictions.
